# Carbohydrate antigen 125 supplements carbohydrate antigen 19-9 for the prediction of invasive intraductal papillary mucinous neoplasms of the pancreas

**DOI:** 10.1186/s12957-022-02720-0

**Published:** 2022-09-26

**Authors:** Yunzhen Qian, Yitao Gong, Guopei Luo, Yu Liu, Ruijie Wang, Xuan Zou, Shengming Deng, Xuan Lin, Yusheng Chen, Xu Wang, Xianjun Yu, He Cheng, Chen Liu

**Affiliations:** 1 Department of Pancreatic Surgery, Fudan University Shanghai Cancer Center, Shanghai, 200032 China; 2 Department of Oncology, Shanghai Medical College, Fudan University, Shanghai, 200032 China; 3 Shanghai Pancreatic Cancer Institute, Shanghai, 200032 China; 4 Pancreatic Cancer Institute, Fudan University, Shanghai, 200032 China

**Keywords:** Intraductal papillary mucinous neoplasms, Serological biomarkers, Diagnostic indices

## Abstract

**Background:**

Intraductal papillary mucinous neoplasms (IPMNs) are characterized by their abundant mucin production and malignant potential. IPMNs of the pancreas are mainly managed according to their radiographic indications, but this approach lacks accuracy with regard to IPMN grading. Therefore, serological biomarkers such as CA19-9 and CA125 (MUC16) should be employed to assist in predicting the invasiveness of IPMNs.

**Methods:**

We investigated the preoperative serum levels of CA19-9, CA125 and CEA in 381 surgical patients with a definite pathological diagnosis of IPMN from July 2010 to December 2019 at the Shanghai Cancer Center. We calculated the Youden indices of each point on the receiver operating characteristic (ROC) curves to identify the most appropriate cut-off values of CA19-9, CA125 and CEA for recognizing malignant IPMNs. Serological biomarker differences were correlated with clinicopathological features of IPMNs, and diagnostic indices of different scenarios were calculated to find the optimum strategy.

**Results:**

The malignant group had higher serum levels of CA19-9, CA125 and CEA. According to the ROC curves, the cut-off values of CA19-9, CA125 and CEA were readjusted to 38.3 U/ml, 13.4 U/ml and 5.3 μg/L. CA19-9 elevation was significantly associated with vascular invasion and perineural infiltration. CA125 showed good efficacy in predicting invasive IPMN in the CA19-9-negative subgroup.

**Conclusions:**

Serological biomarkers are useful and sensitive indicators for recognizing invasive IPMNs. CA19-9 is the most important diagnostic index among all routinely measured serum biomarkers for differentiating malignant from benign IPMNs. CA19-9 should be combined with CA125 to enable more accurate predictions of IPMN malignancy.

## Introduction

Pancreatic intraductal papillary mucinous neoplasms (IPMNs) are characterized by abundant mucin production, pancreatic ductal dilation and intraductal growth. Patients with IPMNs are generally older in age, with abdominal pain, diarrhoea and weight loss being the major symptoms [1]. Over the past few decades, the incidence of IPMN has increased, and it now constitutes the largest proportion of pancreatic cystic lesions [2, 3]. IPMNs differ in terms of their malignant potential; malignant IPMN is considered a premalignant condition and has the potential to transform into aggressive cancer, so surgical intervention is warranted for patients with these early-stage cancerous lesions. However, for benign IPMNs, lifelong follow-up is recommended rather than surgical intervention [2] because pancreatic surgery is complicated and carries a high risk of complications, especially for lesions located in the head of the pancreas. Therefore, accurate evaluation of IPMN malignancy is important for clinical management.

Currently, IPMNs of the pancreas are managed mainly according to their morphological features, which can be distinguished by radiological examinations such as cross-sectional computerized tomography (CT), magnetic resonance imaging (MRI) and endoscopic ultrasonography (EUS). According to morphological stratification, patients with main pancreatic duct IPMN (MD-IPMN) and mixed-type IPMN (mix-IPMN) are recommended for surgery [4]. With the popularization of radiological examinations, there has been an increasing number of incidentally diagnosed IPMNs, which can be treated with different types of surgeries. However, the proportion of malignant IPMNs that are resected among all IPMN resection surgeries is decreasing [5], which indicates that the efficacy of radiological examination is unsatisfactory and leads to unnecessary surgery. Therefore, other indications, such as serological biomarkers, should be taken into consideration for a more accurate assessment of the malignant potential of IPMNs [6].

The routinely measured serological biomarkers for pancreatic cancerous disease include carbohydrate antigen 19-9 (CA19-9), carbohydrate antigen 125 (CA125) and carcinoembryonic antigen (CEA). CA19-9, also known as sialyl Lewis A, is an important serological biomarker for pancreatic diseases and an indicator of aberrant glycosylation [7]. CA19-9 is commonly used to predict the malignancy of various kinds of pancreatic lesions, such as pancreatic ductal adenocarcinoma [8] and pancreatic mucinous cystic neoplasms [9]. CA125, also known as MUC16, is a membrane-spanning glycoprotein secreted into the bloodstream by epithelial cells. CA125 is mostly used for gynaecological diseases. However, pancreatic IPMNs have extensive mucin production; hence, there may also be a possible correlation between serum MUC16 levels and the biological behaviour of IPMNs. CEA was first identified in colon cancer and has been widely used for the diagnosis of gastrointestinal neoplasms [10, 11]. The combination of multiple biomarkers can overcome the limitations related to the use of a single biomarker. For instance, the possibility of false-negative results is the major limitation of CA19-9, but CA125 and CEA show greater efficacy in diagnosing CA19-9-negative pancreatic cancer [12, 13].

In this study, we investigated whether CA125 or CEA could be combined with CA19-9 to distinguish high-grade IPMN from indolent dysplasia. We readjusted the cut-off values of the three biomarkers and compared the diagnostic efficacy of various combinations. In addition, we examined the CA19-9-negative IPMN patient subgroup and analysed the difference in serological biomarker levels. To the best of our knowledge, this is the first article to propose optimum cut-off values for CA19-9, CA125 and CEA to predict invasive pancreatic IPMNs and to evaluate different combinations of these biomarkers.

## Materials and methods

### Design and patients

This retrospective study, consisting of 381 patients with a definite postoperative pathological diagnosis of pancreatic IPMN from July 2010 to December 2019, was conducted at the Shanghai Cancer Center, Fudan University, China. All patients underwent radical surgical resection and subsequent treatment according to the consensus guidelines published by the International Association of Pancreatology (IAP) [4]. The exclusion criteria were as follows: (1) patients with secondary malignancies or multiple primary malignancies, (2) patients with benign oncologic diseases other than pancreatic IPMN, (3) patients with serum bilirubin greater than 34.2 μmol/L, (4) patients with haematological disorder and (5) patients with inflammatory diseases.

Baseline clinical information, including sex and age, was collected from the patients’ medical records. Preoperative serum levels of CA19-9, CEA and CA125 were measured within 2 weeks before surgery. Postoperative pathological reports, including tumour grade, tumour behaviour and tumour location, were acquired from the Fudan University Shanghai Cancer Center. Tumour grade was assessed according to the fifth edition of the WHO Classification of Tumours [14] and was reviewed by two expert pathologists. IPMNs were classified into four pathological grades: invasive carcinoma, high-grade dysplasia, moderate-grade dysplasia and low-grade dysplasia. Preoperative CT, MRI and EUS examination results were reviewed to determine whether patients had a dilated pancreatic duct.

The major objective of this research is to assess the clinical significance of serum biomarkers for predicting IPMNs with malignant potential and to determine the optimal serum biomarker combination for guiding future clinical practice. Patients with invasive carcinoma and high-grade dysplasia were integrated into the malignant IPMN subgroup, and patients with moderate-grade and low-grade dysplasia were integrated into the benign IPMN subgroup. The following analysis of IPMN malignancy was performed on the basis of this classification.

Additional objectives included identifying optimal cut-off values and investigating the associations between serum biomarker levels and clinicopathological features. This study was approved by the Ethics Board of Shanghai Cancer Center, Fudan University, and all 381 patients involved in this study provided written informed consent for the use of their personal data for research purposes during their hospitalization.

### Statistical analysis

The comparison of serum biomarker levels of each subgroup was based on the mean values, and Welch’s *t*-test was employed to analyse the significant difference. The dispersion distribution of tumour markers is shown in scatter plots. Pearson’s χ^2^ test and Fisher’s exact test were used to analyse the correlations between serum biomarker levels and clinicopathological characteristics. All statistical analyses were performed using SPSS 26.0 software (SPSS, Inc., Chicago, IL). A *p*-value < 0.05 was considered statistically significant, and all *p*-values were two sided.

## Results

### Baseline characteristics

A total of 381 patients had a pathological diagnosis of IPMN, including 215 male patients and 166 female patients. Their ages ranged from 31 to 84 (median age, 62.4) years old. Of these patients, 228 had lesions located in the head of the pancreas (59.8%), 119 had lesions located in the pancreatic body or tail (31.2%) and the remaining patients did not have a definite lesion location (either multiple IPMN lesions or a large IPMN that covered the pancreatic head, body and tail).

With regard to histological grade, 118 patients had invasive carcinoma (31.0%), 17 patients had high-grade dysplasia (4.5%) and the remaining patients had low- or moderate-grade dysplasia (64.6%). According to the pathological reports, 190 patients had a concrete description of peri-lesion vascular and neural status (135 malignant and 55 benign samples), 5 had vascular invasion (2.6%) and 11 had perineural infiltration (5.8%).

Regarding preoperative serum biomarkers, 94 patients had elevated CA19-9 levels (24.7%, cut-off value = 37 U/ml), 9 patients had elevated CA125 levels (2.36%, cut-off value = 35 U/ml) and 48 patients had elevated CEA levels (12.6%, cut-off value = 5.3 μg/L).

### Value of CA19-9, CA125 and CEA in predicting invasive IPMN

**Fig. 1:**
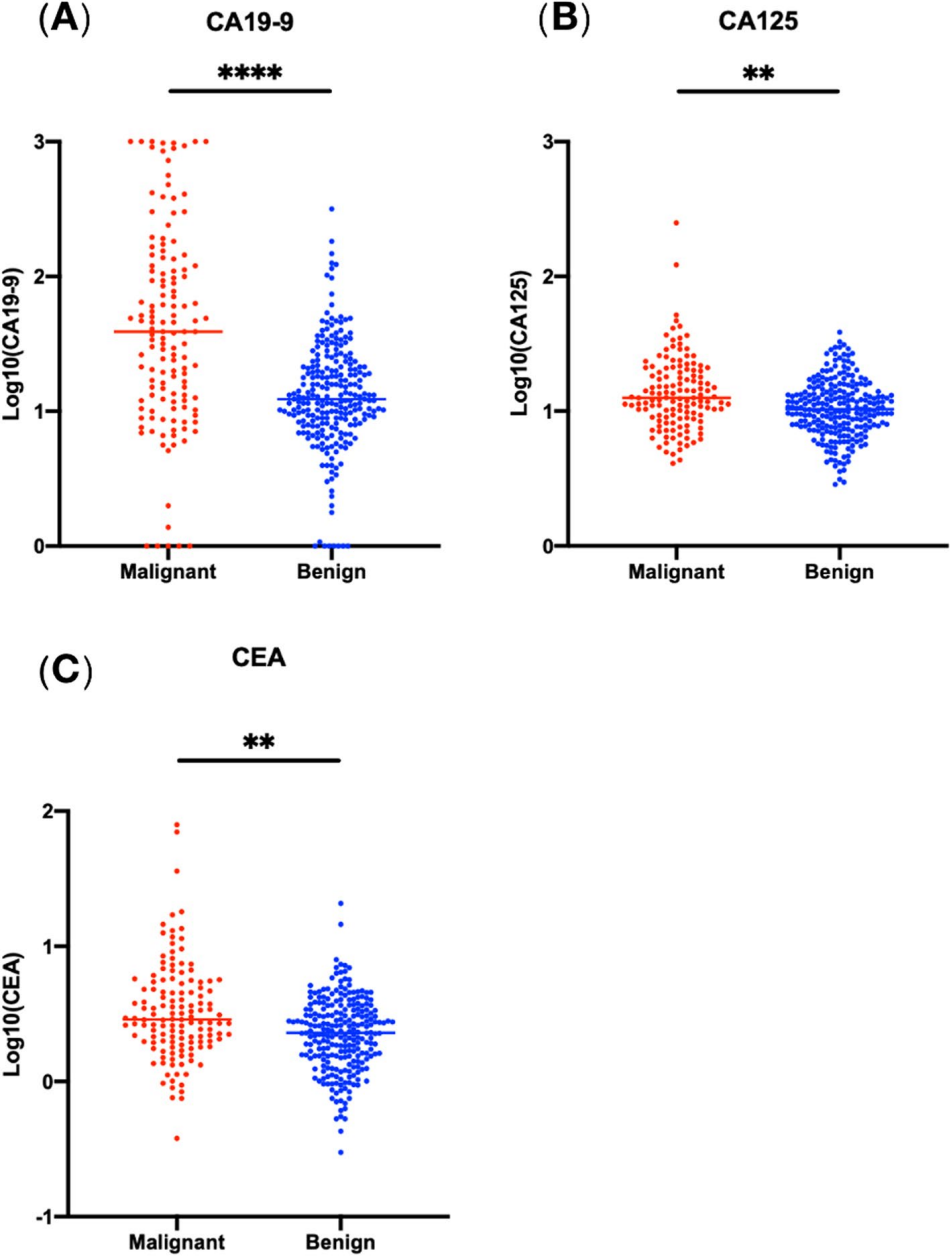
Serum levels of CA19-9, CA125 and CEA in relation to the histological grade of IPMN. Comparisons between different histological grades of IPMN and CA19-9 levels (**A**), CA125 levels (**B**) and CEA levels (**C**). ****p < 0.0001, **p < 0.01, *p*-values derived from Welch’s *t*-test. CA19-9, carbohydrate antigen 19-9; CA125, carbohydrate antigen 125; CEA, carcinoembryonic antigen; IPMN, intraductal papillary mucinous neoplasm

The malignant and benign IPMN subgroups differed in terms of their serum biomarker levels, with the invasive IPMN subgroup having higher CA19-9 levels (149.4 vs. 20.4 U/ml, *p* < 0.0001), higher CA125 levels (17.6 vs. 11.8 U/ml, *p* = 0.0056) and higher CEA levels (5.27 vs. 2.71 μg/L, *p* = 0.0026) (Fig. 1).

Preoperative serum CA19-9, CA125 and CEA levels showed good efficacy in predicting the risk of IPMN malignancy when applied with classical cut-off values (37 U/ml for CA19-9, 35 U/ml for CA125 and 5.3 μg/L for CEA), showing specificities of 90.7%, 99.6% and 94.3%, respectively, but their sensitivities were 51.1%, 5.9% and 25.2%, respectively. Although the specificity was acceptable, the sensitivity was less than satisfactory. To improve the limited diagnostic efficacy of serological biomarkers in recognizing malignant IPMNs, their cut-off values should be readjusted.

### New cut-off values calculated according to the ROC curves

**Fig. 2:**
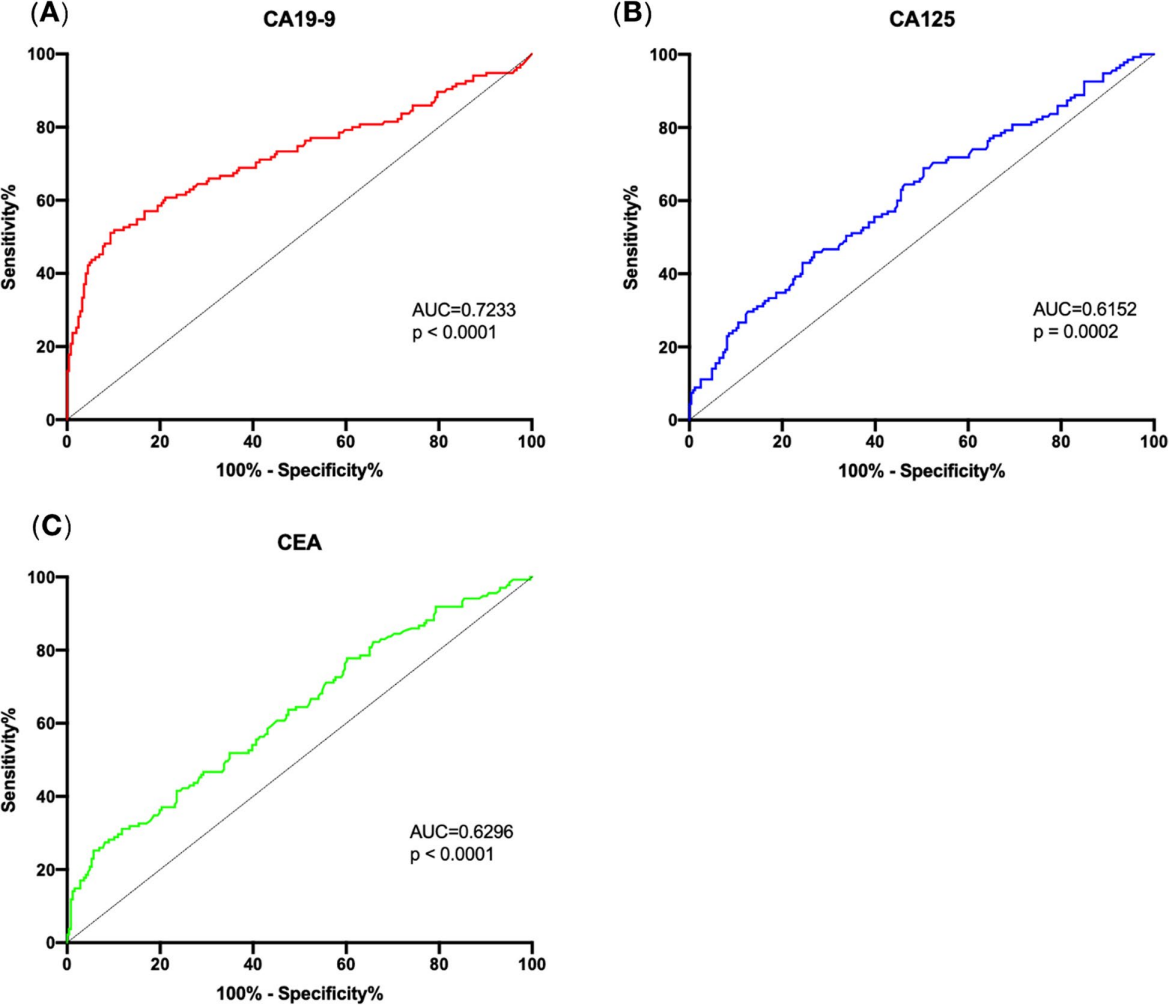
Receiver operating characteristic curves for serum levels of CA19-9, CA125 and CEA. Receiver operating characteristic curves of CA19-9 (**A**), CA125 (**B**) and CEA (**C**) for predicting malignant IPMNs. AUC, area under curve; CA19-9, carbohydrate antigen 19-9; CA125, carbohydrate antigen 125; CEA, carcinoembryonic antigen; IPMN, intraductal papillary mucinous neoplasm

ROC curves were generated for CA19-9, CA125 and CEA, and the areas under the curve (AUCs) of the ROC curves were 0.723, 0.615 and 0.630 (Fig. 2), respectively. CA19-9 was considered to be the centrally used serum biomarker for predicting invasive IPMN because it had the largest AUC.

The Youden indices of each point on the ROC curves were calculated to determine the most appropriate cut-off values, and the adjusted values for CA19-9, CA125 and CEA were 38.33 U/ml (sensitivity = 51.1%, specificity = 90.7%), 13.47 U/ml (sensitivity = 45.9%, specificity = 73.2%) and 5.25 μg/L (sensitivity = 25.2%, specificity = 94.3%), respectively. The readjusted cut-off values of CA19-9 and CEA were similar to the classic cut-off values, but the readjusted cut-off value of CA125 was greatly different from its original cut-off value. We used these new cut-off values for the following analysis.

All three serum biomarkers showed good efficacy in distinguishing malignant IPMNs with the adjusted cut-off values (Tables 1, 2 and 3, *p* < 0.001). In addition, all three biomarkers were correlated with pancreatic ductal dilation, suggesting that serological alterations were associated with radiological indications.

**Table 1:** Characteristics of IPMN patients stratified by CA19-9

Variables	CA19-9 ≤ 38.3 U/ml	CA19-9 > 38.3 U/ml	***p***-value
Age, *n* (%), y			0.229
< 60	98 (33.9%)	25 (27.2%)	
≥ 60	191 (66.1%)	67 (72.8%)	
Sex, (*n*%)			0.235
Male	168 (58.1%)	47 (51.1%)	
Female	121 (41.9%)	45 (48.9%)	
Location, (*n*%)			0.572
Head	172 (64.9%)	56 (68.3%)	
Body and tail	93 (35.1%)	26 (31.7%)	
Tumour grade, (*n*%)			< 0.001
Low and moderate	223 (77.2%)	23 (25.0%)	
High and carcinoma	66 (22.8%)	69 (75.0%)	
Vascular invasion, (*n*%)			0.005
No	153 (99.4%)	32 (88.9%)	
Yes	1 (0.6%)	4 (11.1%)	
Perineural infiltration, (*n*%)			0.036
No	148 (96.1%)	31 (86.1%)	
Yes	6 (3.9%)	5 (13.9%)	
Ductal dilation, (*n*%)			0.002
No	200 (69.2%)	47 (51.1%)	
Yes	89 (30.8%)	45 (48.9%)

**Table 2:** Characteristics of IPMN patients stratified by CA125

Variables	CA125 ≤ 13.4 U/ml	CA125 > 13.4 U/ml	***p***-Value
Age, *n*(%),y			0.940
< 60	82 (32.4%)	41 (32.0%)	
≥ 60	171 (67.6%)	87 (68.0%)	
Sex, (*n*%)			0.043
Male	152 (60.1%)	63 (49.2%)	
Female	101 (39.9%)	65 (50.8%)	
Location, (*n*%)			0.324
Head	147 (63.9%)	81 (69.2%)	
Body and tail	83 (36.1%)	36 (30.8%)	
Tumour grade, (*n*%)			< 0.001
Low and moderate	180 (71.1%)	66 (51.6%)	
High and carcinoma	73 (28.9%)	62 (48.4%)	
Vascular invasion, (*n*%)			0.335
No	125 (98.4%)	60 (95.2%)	
Yes	2 (1.6%)	3 (4.8%)	
Perineural infiltration, (*n*%)			0.510
No	121 (95.3%)	58 (92.1%)	
Yes	6 (4.7%)	5 (7.9%)	
Ductal dilation, (*n*%)			0.013
No	175 (69.2%)	72 (56.3%)	
Yes	78 (30.8%)	56 (43.8%)

**Table 3:** Characteristics of IPMN patients stratified by CEA

Variables	CEA ≤ 5.3 ug/L	CEA > 5.3 ug/L	***p***-Value
Age, *n*(%), y			0.248
< 60	111 (33.3%)	12 (25.0%)	
≥ 60	222 (66.7%)	36 (75.0%)	
Sex, (*n*%)			0.978
Male	188 (56.5%)	27 (56.3%)	
Female	145 (43.5%)	21 (43.8%)	
Location, (*n*%)			0.238
Head	197 (64.6%)	31 (73.8%)	
Body and tail	108 (35.4%)	11 (26.2%)	
Tumour grade, (*n*%)			< 0.001
Low and moderate	232 (69.7%)	14 (29.2%)	
High and carcinoma	101 (30.3%)	34 (70.8%)	
Vascular invasion, (*n*%)			0.121
No	163 (98.2%)	22 (91.7%)	
Yes	3 (1.8%)	2 (8.3%)	
Perineural infiltration, (*n*%)			0.035
No	159 (95.8%)	20 (83.3%)	
Yes	7 (4.2%)	4 (16.7%)	
Ductal dilation, (*n*%)			< 0.001
No	231 (69.4%)	16 (33.3%)	
Yes	102 (30.6%)	32 (66.7%)	

In addition, CA125 was more likely to be elevated in female patients than in male patients (39.2% vs. 29.3%, *p* = 0.043). CA19-9 elevation was correlated with vascular invasion (*OR* = 19.13, *p* = 0.005) and perineural infiltration (*OR* = 3.98, *p* = 0.036). CEA elevation was correlated with perineural infiltration (*OR* = 4.54, *p* = 0.035).

### Combination of CA19-9 with CA125 or CEA

To overcome the limitations related to the use of a single serum biomarker, we made attempts to combine CA19-9 with CA125 or CEA. There was no doubt that combining more biomarkers into a panel results in greater sensitivity, but to ensure that the diagnostic panel is cost-effective, we aimed to use as few biomarkers as possible to supplement CA19-9. Considering the new cut-off value of CA125 was largely different from the classical applied cut-off value of CA125, the two cut-off values of CA125 were thereby both analysed if they could supplement CA19-9 (Table 4).

**Table 4:** Diagnostic indices for CA19-9, CA125, CEA and their combination

	CA19-9	CA125	CA125	CEA	CA19-9 and/or CA125 positive	CA19-9 and/or CA125 positive	CA19-9 and/or CEA positive	CA19-9 and/or CA125 and/or CEA positive	CA19-9 and/or CA125 and/or CEA positive
Cutoff value	38.3 U/mL	13.4 U/mL	35 U/mL	5.3 ug/l	38.3 U/mL and 13.4 U/mL	38.3 U/mL and 35 U/mL	38.3 U/mL and 5.3 ug/l	38.3 U/mL and 13.4 U/mL and 5.3 ug/l	38.3 U/mL and 35 U/mL and 5.3 ug/l
Sensitivity	**51.1%**	45.9%	5.9%	25.2%	**68.9%**	52.6%	59.3%	73.3%	60.0%
Specificity	90.7%	73.2%	**99.6%**	94.3%	68.7%	**90.7%**	85.8%	65.4%	85.8%
Positive predictive value	75.0%	48.4%	**88.9**%	70.8%	54.7%	75.5%	69.6%	53.8%	69.8%
Negative predictive value	**77.2%**	71.1%	65.9%	69.6%	**80.1%**	77.7%	79.3%	81.7%	79.6%
Accuracy	**76.6%**	63.5%	66.4%	69.8%	68.8%	**77.2%**	76.4%	68.2%	**76.6%**
OR	10.14	2.32	15.43	5.58	4.86	10.76	8.77	5.21	9.04

*OR*, odds ratio

The combination of CA19-9 and CA125 (CA19-9 > 38.3 U/ml or CA125 > 35 U/ml) was found to comprehensively improve the diagnostic indices. Other combination strategies, however, did not show overall superiority over the use of CA19-9 alone. The combination of CA19-9 and CA125 (CA19-9 > 38.3 U/ml or CA125 > 35 U/ml) had the best positive predictive value, 77.2%, and the combination of CA19-9 and CA125 with the adjusted cut-off values (CA19-9 > 38.3 U/ml or CA125 > 13.4 U/ml) had the best negative predictive value, 80.1%. Considering the poor prognosis of malignant pancreatic lesions and the major limitations of the use of CA19-9, we speculated that the combination of CA19-9 and CA125 (CA19-9 > 38.3 U/ml or CA125 > 13.4 U/ml) would have clinical benefit for identifying invasive IPMNs (sensitivity = 68.9%) and acceptable accuracy (specificity = 68.9%, accuracy = 68.8%).

### Efficacy of CA125 and CEA in identifying invasive IPMN in the CA19-9-negative subgroup

Although CA19-9 is considered to be the most commonly used biomarker for predicting malignant IPMN, we found that nearly half of invasive IPMNs could not be distinguished by CA19-9 alone (sensitivity = 51.1%). There was a considerable population of Lewis-negative patients who had deficiencies in biosynthesizing or secreting CA19-9 [15]. Therefore, it is necessary to identify suitable biomarkers for predicting invasive IPMN in CA19-9-negative patients.

**Fig. 3:**
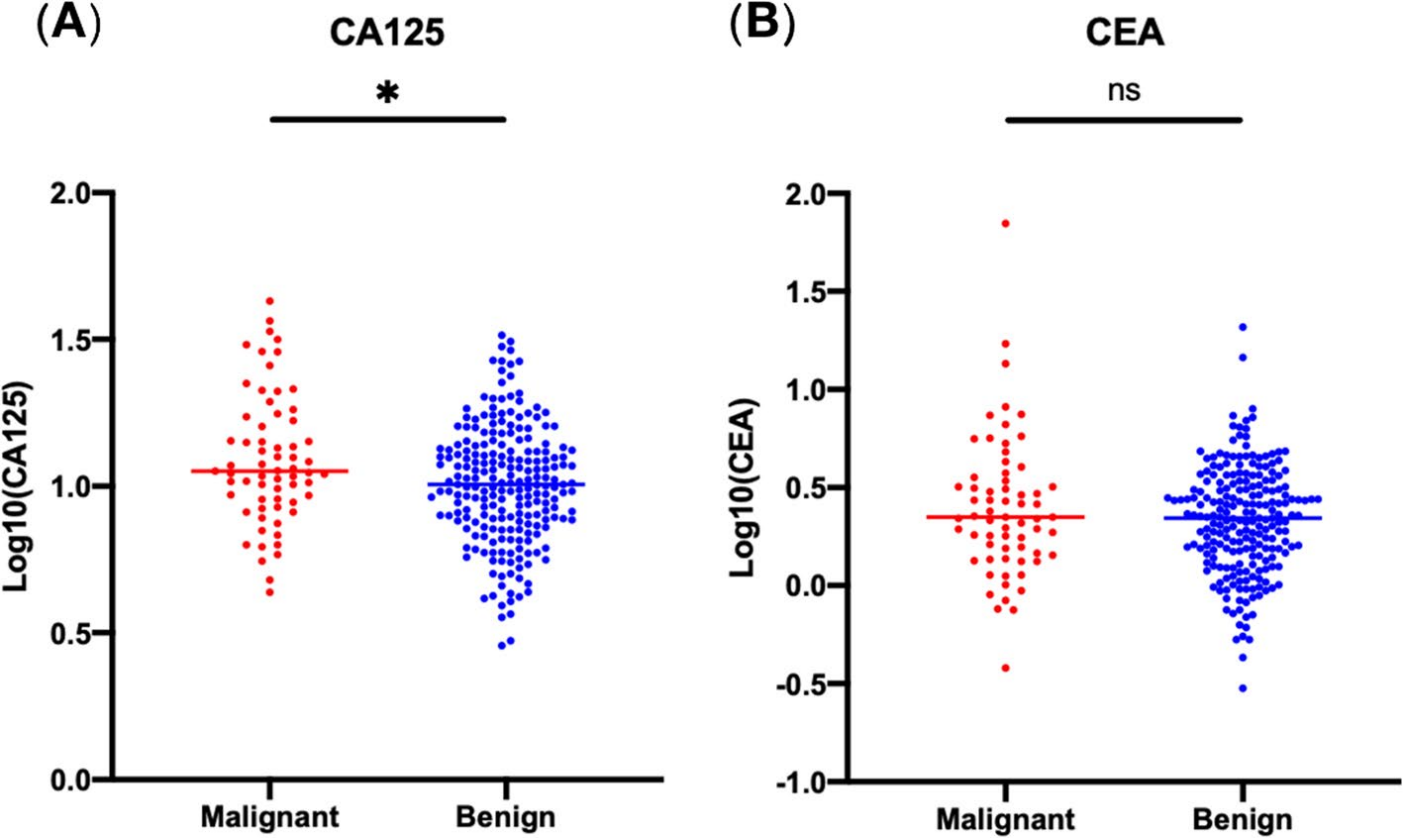
Serum levels of CA125 and CEA in relation to the histological grade of IPMN in CA19-9-negative patients. Comparisons between different histological grades of IPMN without CA19-9 elevation and CEA levels (**A**) and CA125 levels (**B**).*p < 0.05, ns, not significant, *p*-values derived from Welch’s *t*-test. CA19-9, carbohydrate antigen 19-9; CA125, carbohydrate antigen 125; CEA, carcinoembryonic antigen; IPMN, intraductal papillary mucinous neoplasm

Regarding CA19-9-negative IPMN patients (*n* = 286), 27.3% of patients had elevated serum CA125 levels (cut-off value = 13.4 U/ml), and 8.0% of patients had elevated serum CEA levels (cut-off value = 5.3 μg/L). The malignant subgroup had higher CA125 levels (14.08 vs. 11.24 U/ml, *p* = 0.011) and higher CEA levels (4.11 vs. 2.63 μg/L, *p* = 0.183, Fig. 3).

**Fig. 4:**
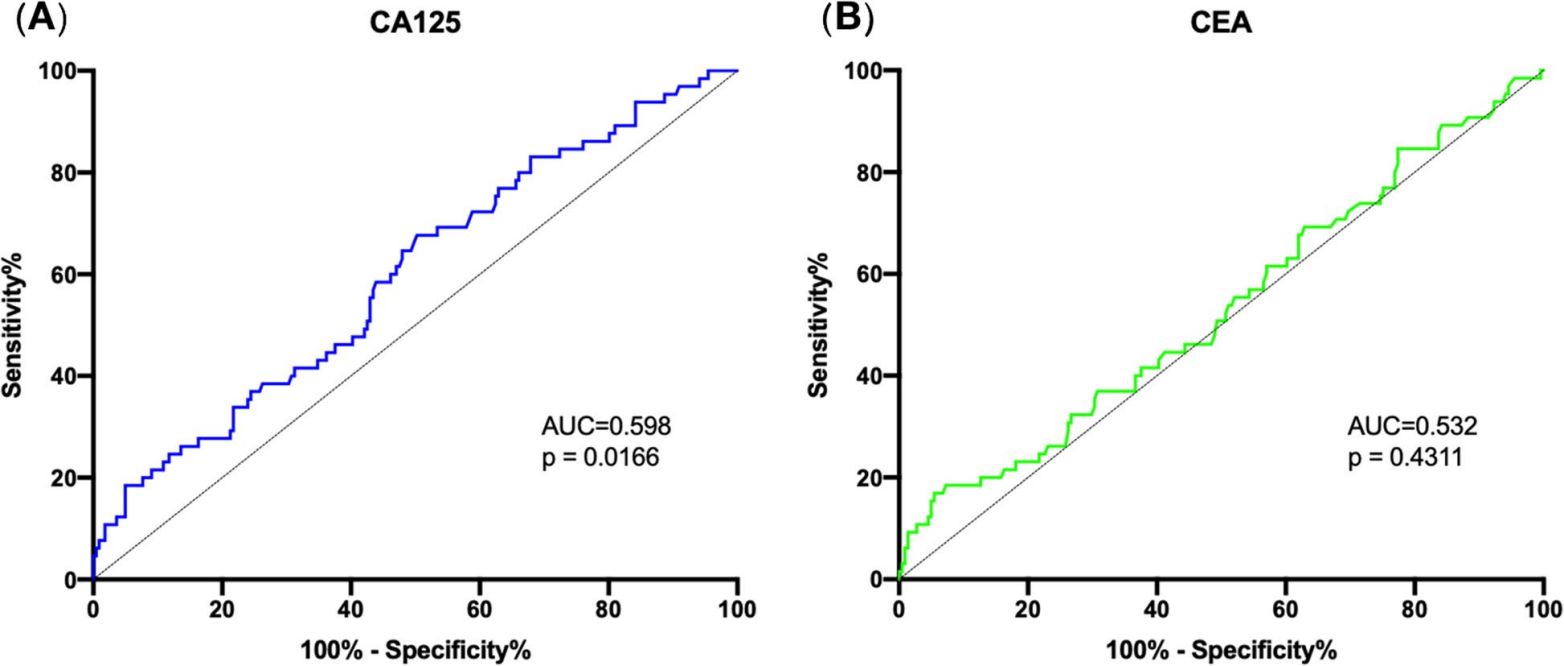
Receiver operating characteristic curves for CA125 and CEA in CA19-9-negative patients. Receiver operating characteristic curves of CA125 (**A**) and CEA (**B**) for predicting malignant IPMNs in CA19-9-negative patients. AUC, area under curve; CA19-9, carbohydrate antigen 19-9; CA125, carbohydrate antigen 125; CEA, carcinoembryonic antigen; IPMN, intraductal papillary mucinous neoplasm; ROC, receiver operating characteristic curves

ROC curves were generated to illustrate the efficacy of CEA and CA125 in recognizing invasive IPMNs in CA19-9-negative patients (Fig. 4). The AUCs of CA125 and CEA were 0.598 and 0.532, respectively (*p* = 0.0166, *p* = 0.4311). Furthermore, according to the Youden index, the optimum cut-off values of CA125 and CEA in predicting CA19-9-negative invasive IPMNs were 10.1 U/ml (sensitivity = 67.7%, specificity = 49.8%) and 5.3 μg/L (sensitivity = 16.9%, specificity = 94.6%), respectively. Therefore, CA125 was found to be superior to CEA in predicting CA19-9-negative invasive IPMNs.

Briefly, it is recommended that CA19-9 be measured centrally, and in CA19-9-negative patients, CA125 (cut-off value = 10.1 U/ml) should take priority for identifying invasive IPMNs.

## Discussion

In this retrospective study, we analysed serum biomarkers of 381 IPMN patients and found that patients with malignant IPMNs had higher serum CA19-9, CA125 and CEA levels. CA19-9 showed the best diagnostic efficacy among all routinely tested biomarkers and was accordingly considered to be centrally used for detecting malignant IPMNs. In addition, we readjusted the cut-off values of all three biomarkers and found that a lower cut-off value (13.4 U/ml) should be adopted for CA125 to accurately identify pancreatic malignant IPMNs. With this adjusted cut-off value, CA125 can serve as a supplement to CA19-9 to overcome its limited sensitivity. Interestingly, we also found that CA125 was more likely to be elevated in female patients.

We also found association between serological abnormalities and pancreatic ductal dilation and IPMN malignant biological behaviour, i.e. vascular invasion and perineural infiltration. According to previous reports, vascular invasion and perineural infiltration were more likely to be presented in invasive IPMN [16] and denoted poor survival of IPMN [17, 18]. Therefore, further study investigating whether serological biomarkers could predict IPMN prognosis is warranted.

As mentioned before, a high false-negative rate has restricted the application of CA19-9 as a biomarker in clinical practice. Previous studies have investigated the efficacy of CA19-9 in distinguishing malignant and benign IPMNs, and the sensitivity of CA19-9 was reported to range from 40.8 to 74.0% [19, 20]. The unsatisfactory sensitivity of CA19-9 can be partially attributed to the heterogeneity of the Lewis blood group. Approximately, 5–7% of the population belongs to the Lewis^a-b^ subgroup, who do not produce and release CA19-9 into blood owing to various metabolic disorders. For example, patients with FUT3 deficiency are not able to synthesize CA19-9; in patients with FUT2 overexpression, their cells consume the substrate for CA19-9 synthesis, and patients with secretor 21 deficiency are not able to secrete CA19-9 into serum [21]. To overcome this issue, it is necessary to combine other biomarkers with CA19-9 in clinical practice. This study investigated routinely measured serum biomarkers and revealed that CA125 measurement can supplement CA19-9 and increase the diagnostic accuracy of predicting IPMN malignancy. The combination of CA125 and CA19-9 showed improved diagnostic sensitivity in recognizing invasive IPMNs. On the other hand, the diagnostic efficacy of CEA in distinguishing benign and invasive IPMNs of the pancreas was less than satisfactory, and the combination of CEA and CA19-9 did not improve the diagnostic accuracy of these indices [20]. According to the ROC curves, CA125 showed superiority to CEA in terms of supplementing CA19-9. Furthermore, in the CA19-9-negative group of patients in the malignant subgroup, serum CA125 levels were significantly elevated, whereas CEA levels were not.

Another limitation of CA19-9 application is the possibility of false-positive results. CA19-9 elevation also appears in inflammatory diseases and non-pancreatic cancers such as pancreatitis, cholestasis and cholangiocarcinoma. Because CA19-9 is also excreted by normal biliary epithelial cells, CA19-9 has a significantly higher serum level in patients with bile tract obstruction [22]. This study had excluded patients with elevated bilirubin to avoid its confounding. Of all investigated 381 IPMN patients, 11 patients were radiologically diagnosed as having biliary tract obstruction with an average CA19-9 level of 200.8 U/ml. Therefore, readjustment of the cut-off value of CA19-9 according to patients’ serum bilirubin levels and biliary obstruction status will help to improve the efficacy of CA19-9 in future studies. Multivariate analysis including serum bilirubin level and other false-positive factors is also warranted. In addition, efforts have been made to identify more accurate serum metabolites [23]. Nonetheless, the reported specificity of CA19-9 in distinguishing malignant IPMNs ranges from 84.5 to 85.9% [19, 20], which is acceptable.

The major hindrance of the use of CA125 and CEA is their inappropriate cut-off values. First, CA125 and CEA are commonly used for gynaecological diseases and colorectal diseases, and pancreatic IPMN has its own specific metabolic features, i.e. abundant mucin production, so the original commonly used cut-off values are not suitable. Second, IPMN is a precancerous disease, so its pathological and serological alterations are not completely consistent with those observed in cancerous diseases. Thus, more sensitive cut-off values are needed for this condition. This study also found that a lower cut-off value should be adopted for CA125 (MUC16) for the diagnosis of malignant pancreatic IPMNs.

Remarkably, these serological markers are not simply indirect indicators but are also culprits in pancreatic disease (Table 5). For example, CA19-9 modifies the matricellular protein fibulin-3, increases its interaction with EGF, and cooperates with the Kras G12D oncogene to promote pancreatic tumorigenesis [24]. CA125 is involved in cell signalling through the phosphorylation of its C-terminal domain and has a potential pro-metastatic role in cancer cells [25]. CEA functions as a homotypic intercellular adhesion molecule and mediates tumour invasion and metastasis. Therefore, we speculate that the elevation of these serum biomarkers in IPMN patients not only indicates the malignancy of IPMN but also contributes to the progression of IPMN into more invasive pathological subtypes, thus correlating with IPMN patient survival. A previous investigation confirmed that CA19-9 elevation is correlated with worse overall survival and disease-free survival in IPMN [19].

**Table 5:** Biological characteristics of CA19-9, CA125 and CEA

	CA19-9	CA125	CEA
Structure	Tetrasaccharide	Mucin (MUC16)	Glycoproteins
Biosynthesis	Series of glycosyltransferation	Coding genes transcription and translation	
Most common application	Pancreatic cancer	Ovarian cancer	Colorectal cancer
Cellular location	Various protein carried	Membrane tethered	Cell surface oriented
Characteristic action modes	1) Glycosylating proteins 2) Binding to E-selectin 3) Promoting angiogenesis	1) Reacting with apical surface of epithelial cells 2) Binding to L-selectin and E-selectin 2) C-terminal domain phosphorylating for cell signalling	1) Homotypic adhering 2) Blocking terminal myogenic differentiation
Reasons for false negative	Biosynthesis failure owing to absence of crucial glycosyltransferase	1) Coding genes’ mutation 2) Poor blood circulation in tumour location	
Reasons for false positive	1) Bile duct obstruction 2) Liver damage 2) Pancreatitis	1) Menstruation 2) Pelvic inflammatory disease 2) Endometriosis	1) Inflammatory bowel diseases 2) Smoking 2) Colon polyps

In addition to carbohydrate antigens, many other serological indices were also applied in the appraisal of IPMN malignancy, such as fibrinogen [26], lipase and amylase [27, 28]. Furthermore, newly discovered biomarkers showed efficacy in detecting and grading IPMNs [29]. A 9-miRNA signature in pancreatic cyst fluid distinguished malignant IPMN with a specificity of 100% and a sensitivity of 89% [30]. Certain circulating microRNAs were significantly elevated in patients with malignant IPMN, such as miRNA-483-3p, miRNA-21 [31], miRNA-1290 [32] and miRNA-223 [33]. Circulating epithelial cells [34], circulating cell-free DNA [35, 36] and circulating extracellular vehicles [37] had implications for IPMN risk stratification as well. Moreover, IPMN malignant conversion was accompanied by serum antibody levels alteration, such as antibody to p53 [38] and antibody to microbes [39]. Although those newly discovered serological biomarkers were not commonly measured in most medical centres, they were very promising tools for managing IPMN (Table 6).

**Table 6:** Circulating biomarkers other than carbohydrate antigens for recognizing malignant IPMNs. Cutoff values were annotated in brackets. The number of malignant IPMN patients and total IPMN patients were displayed in column *investigation sample size*

	Serological biomarkers	Investigation sample size	Sensitivity	Specificity	Reference (PMID)
Classical biomarker	Fibrinogen (4.71 g/L)	21/73	42.9%	88.5%	28131667
	Serum pancreatic enzymes (lipase and amylase)	54/203	Cutoff values did not determined		25239347
		42/142			27394653
Extracellular vesicle	MUC5 (10, DEST assay)	34/123	100%	82%	33301777
MicroRNA	miR-223 (125.0 atto M)	28/36	82.4%	62.9%	25819175
	miR-1290	16/20	Cutoff value did not determined (*AUC* = 0.82)		23697990
	miR-483-3p and miR-21	32/44	Cutoff values did not determined (*AUC* = 0.703 and 0.603, respectively)		25384963
Antibody	CEA (5.0 ng/ml) CA9-9 (37 U/ml) p53 antibody (1.3 U/ml)	73/111	38.4%	81.6%	32541631
	Antibodies to *Fusobacterium nucleatum*	46/91	Cutoff value did not determined		32983143

*DEST* digital extracellular vesicle screening technique

The strength of our research is that we solely focus on pathologically diagnosed IPMN patients, which makes our study sample more homogenous. In addition, we included 381 IPMN patients in our study, which is comparatively large and makes our results very convincing. Moreover, we separately analysed serological biomarker levels in CA19-9-negative IPMN patients to demonstrate the significance of CA125 in these patients. To the best of our knowledge, no previous study has reported on the significance of CA125 in recognizing invasive IPMNs of the pancreas.

The primary limitation of our study is that it is a surgical cohort. In addition, we did not match serological biomarkers according to bilirubin levels or morphological subtypes, which are also risk factors for IPMN [40]. Additionally, there were no data on survival in our study.

## Conclusion

The results of our study demonstrate that CA19-9 is the best serological biomarker to recognize invasive IPMN, and its combination with CA125 helps to overcome the unsatisfactory sensitivity of CA19-9 alone. Therefore, CA19-9 can be centrally measured to predict IPMN malignancy in clinical practice, but it is important to take the elevation of CA125 (cut-off value = 13.4 U/ml) into consideration. Particularly, in Lewis-negative patients, CA125 (cut-off value = 10.1 U/ml) takes priority for distinguishing between the histological subtypes of IPMN.

## Data Availability

The datasets used and analysed during the current study are available from the corresponding author on reasonable request.

## Abbreviations

AUC: Areas under the curve
CA125: Carbohydrate antigen 125
CA19-9: Carbohydrate antigen 19-9
CEA: Carcinoembryonic antigen
CT: Cross-sectional computerized tomography
EUS: Endoscopic ultrasonography
IAP: The International Association of Pancreatology
IPMN: Intraductal papillary mucinous neoplasm
MRI: Magnetic resonance imaging
ROC: Receiver operating characteristic
